# Deciphering Structural Complexity of Brain, Joint, and Muscle Tissues Using Fourier Ptychographic Scattered Light Microscopy

**DOI:** 10.1101/2024.11.28.625428

**Published:** 2024-11-29

**Authors:** Simon E. van Staalduine, Vittorio Bianco, Pietro Ferraro, Miriam Menzel

## Abstract

Fourier Ptychographic Microscopy (FPM) provides high-resolution imaging and morphological information over large fields of view, while Computational Scattered Light Imaging (ComSLI) excels at mapping interwoven fiber organization in unstained tissue sections. This study introduces Fourier Ptychographic Scattered Light Microscopy (FP-SLM), a new multi-modal approach that combines FPM and ComSLI analyses to create both high-resolution phase-contrast images and fiber orientation maps from a single measurement. The method is demonstrated on brain sections (frog, monkey) and sections from thigh muscle and knee (mouse). FP-SLM delivers high-resolution images while revealing fiber organization in nerve, muscle, tendon, cartilage, and bone tissues. The approach is validated by comparing the computed fiber orientations with those derived from structure tensor analysis of the high-resolution images. The comparison shows that FPM and ComSLI are compatible with each other and yield fully consistent results. Remarkably, this combination surpasses the sum of its parts, so that applying ComSLI analysis to FPM recordings and vice-versa outperforms both methods alone. This cross-analysis approach can be retrospectively applied to analyze any existing FPM or ComSLI dataset (acquired with LED array and low numerical aperture), significantly expanding the application range of both techniques and enhancing the study of complex tissue architectures in biomedical research.

## Full Text Availability

The license terms selected by the author(s) for this preprint version do not permit archiving in PMC. The full text is available from the preprint server.

