# Supplementary material for: Deciphering structural complexity of brain, joint, and muscle tissues using Fourier Ptychographic Scattered Light Microscopy
Source: bioRxiv. 2025 Apr 9:2024.11.28.625428. Originally published 2024 Nov 29. Preprint. [Version 2] doi: 10.1101/2024.11.28.625428 (PMC11623658; doi:10.1101/2024.11.28.625428)
Supplement: 1 [file NIHPP2024.11.28.625428V2-supplement-1.pdf]

bioRxiv preprint doi: https://doi.org/10.1101/2024.11.28.625428; this version posted April 9, 2025. The copyright holder for this preprint (which was not certified by peer review) is the author/funder, who has granted bioRxiv a license to display the preprint in perpetuity. It is made available under aCC-BY 4.0 International license.

SUPPORTING INFORMATION

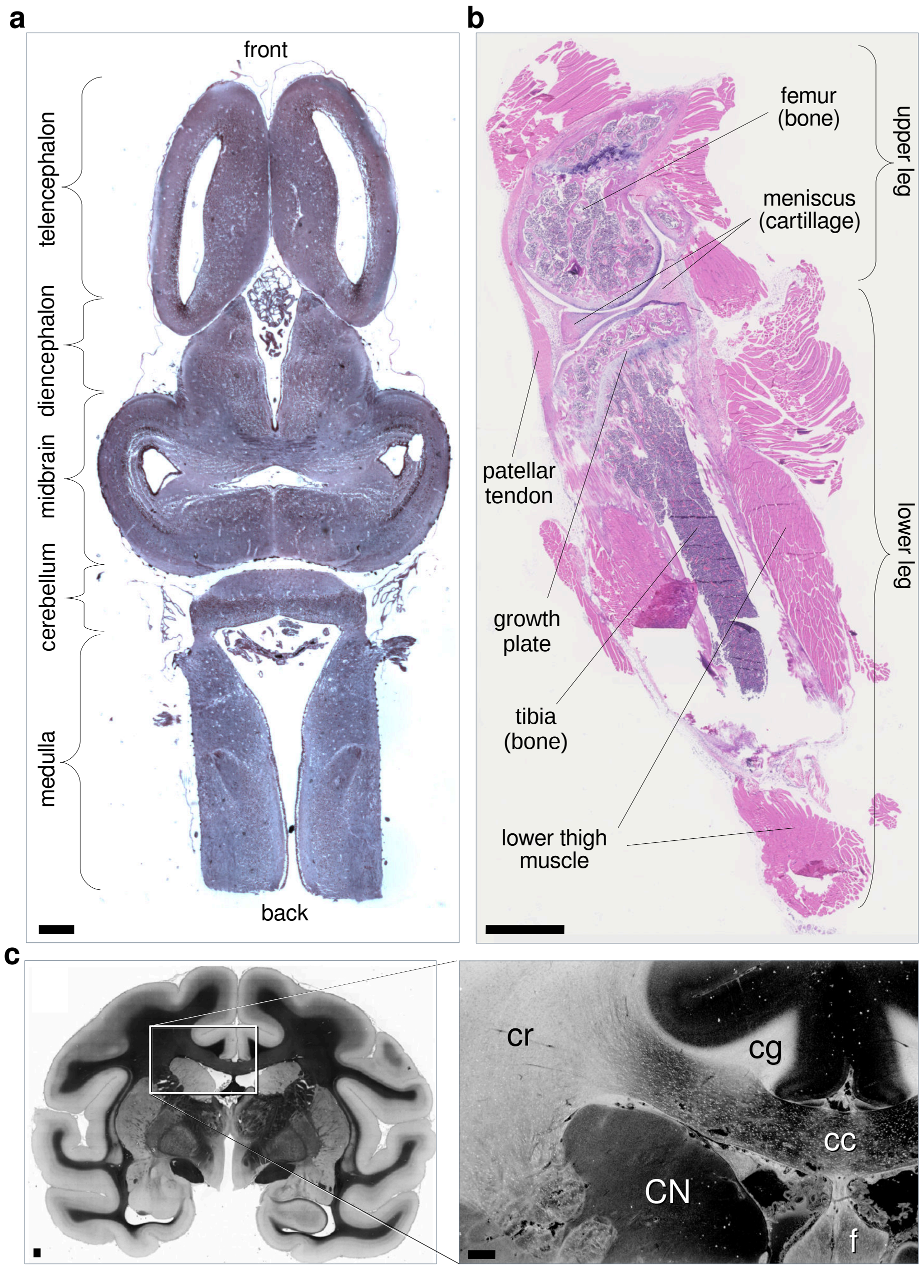

Supplementary Figure 1: Measured samples with anatomical descriptions. a) Horizontal frog tadpole brain section. b) Longitudinal mouse knee section. c) Coronal vervet monkey brain section (bright-field image) with zoomed-in region (average scattering map), cr–corona radiata, cg–cingulum, cc–corpus callosum, f–fornix, CN–caudate nucleus. Scale bars are 1 mm.

bioRxiv preprint doi: https://doi.org/10.1101/2024.11.28.625428; this version posted April 9, 2025. The copyright holder for this preprint (which was not certified by peer review) is the author/funder, who has granted bioRxiv a license to display the preprint in perpetuity. It is made available under aCC-BY 4.0 International license.

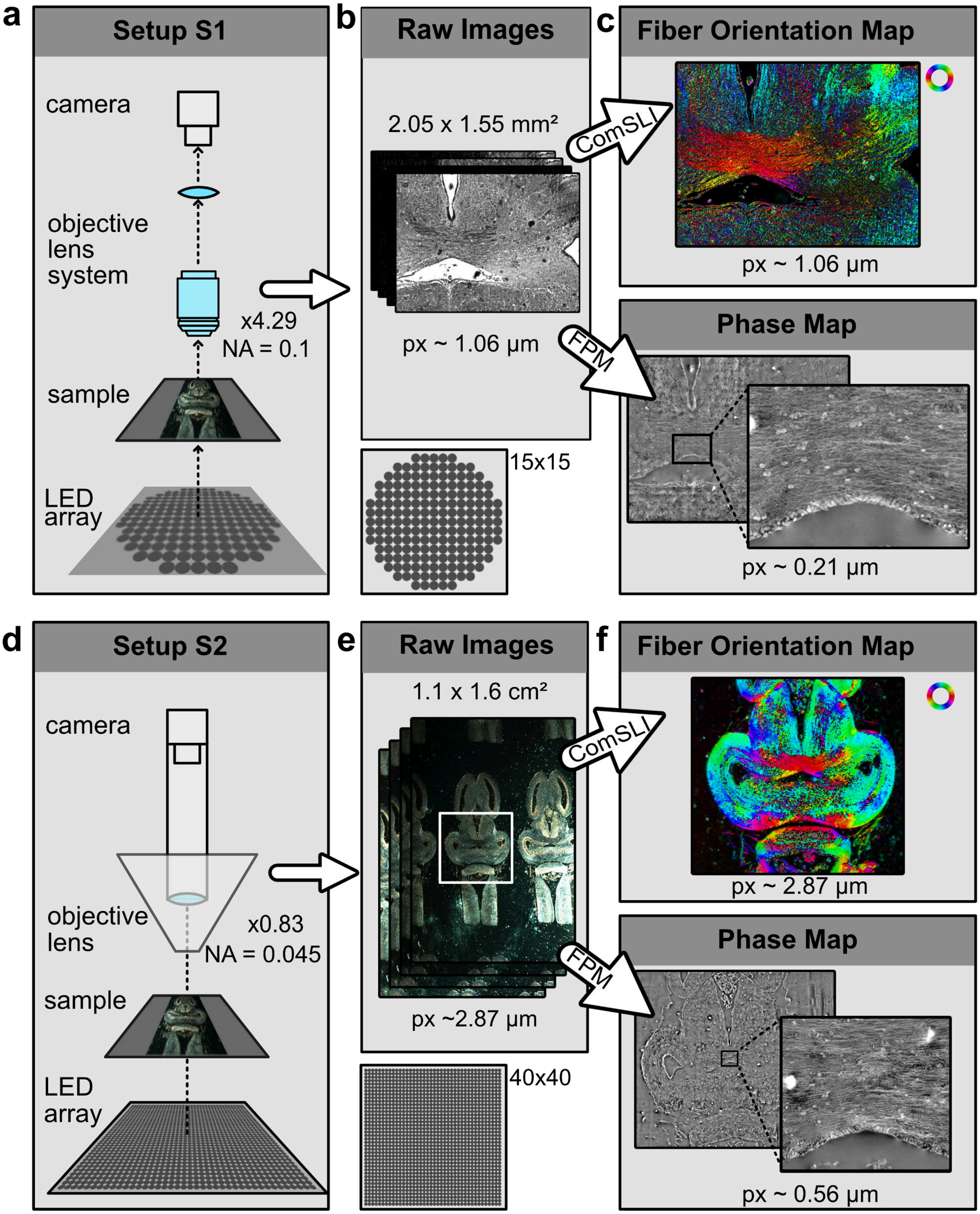

Supplementary Figure 2: Comparison of the two setups used in this study: S1 (originally designed for FPM) and S2 (originally designed for ComSLI). a,d) Sketch of the two setups which employ different LED arrays and optical systems with different numerical apertures (NA) and magnifications. b,e) Image stacks resulting from a measurement with setup S1 and S2, with different fields of view (top) and pixel sizes (bottom). c,f) Corresponding fiber orientation maps (top) and phase maps (bottom) with different pixel sizes (px) obtained from a ComSLI and FPM analysis, respectively, shown for the marked regions.

bioRxiv preprint doi: https://doi.org/10.1101/2024.11.28.625428; this version posted April 9, 2025. The copyright holder for this preprint (which was not certified by peer review) is the author/funder, who has granted bioRxiv a license to display the preprint in perpetuity. It is made available under aCC-BY 4.0 International license.

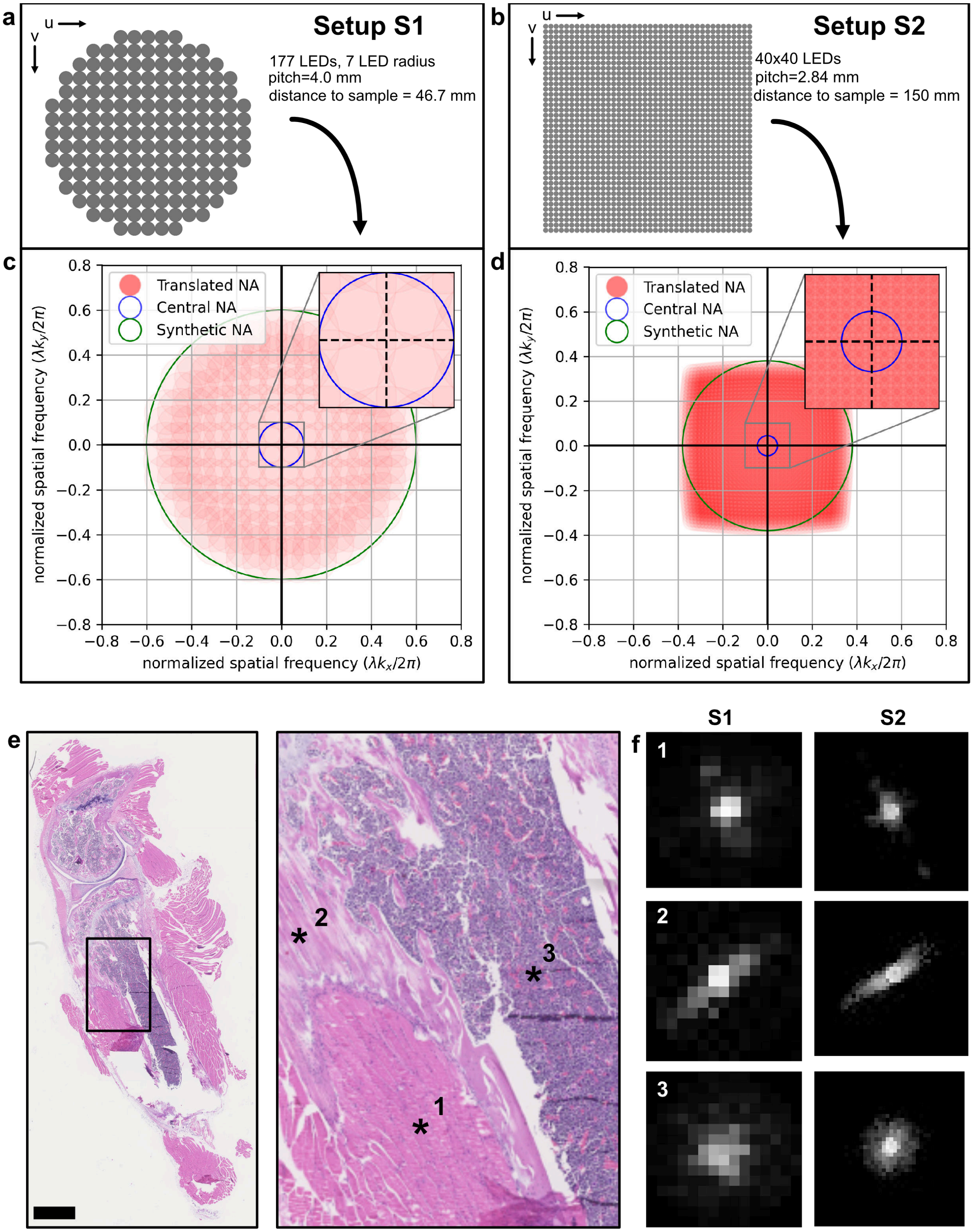

Supplementary Figure 3: Scattering pattern generation. a),b) Layout of the LED arrays used in setup S1 (a) and in setup S2 (b). c),d) Frequency space coverage of the associated LED arrays (red), shown together with the coverage for direct illumination (blue) and synthetic numerical aperture (green). e) Zoom-in of the mouse knee histology section, showing three selected pixels: (1) muscle, (2) tendon, and (3) bone. Scale bar indicates 1 mm. f) Scattering patterns for the three selected pixels, obtained from setup S1 (left) and setup S2 (right). Tendon shows the highest degree of aligned structures (collagen fibers), resulting in a high-intensity line perpendicular to the fiber orientation (2). Bone is less intrinsically oriented and scatters more broadly than the other tissue types (3). Due to the different numbers of LEDs used for the measurements, S1 scattering patterns are more discretized than S2 scattering patterns, but they still correspond to each other.

bioRxiv preprint doi: https://doi.org/10.1101/2024.11.28.625428; this version posted April 9, 2025. The copyright holder for this preprint (which was not certified by peer review) is the author/funder, who has granted bioRxiv a license to display the preprint in perpetuity. It is made available under aCC-BY 4.0 International license.

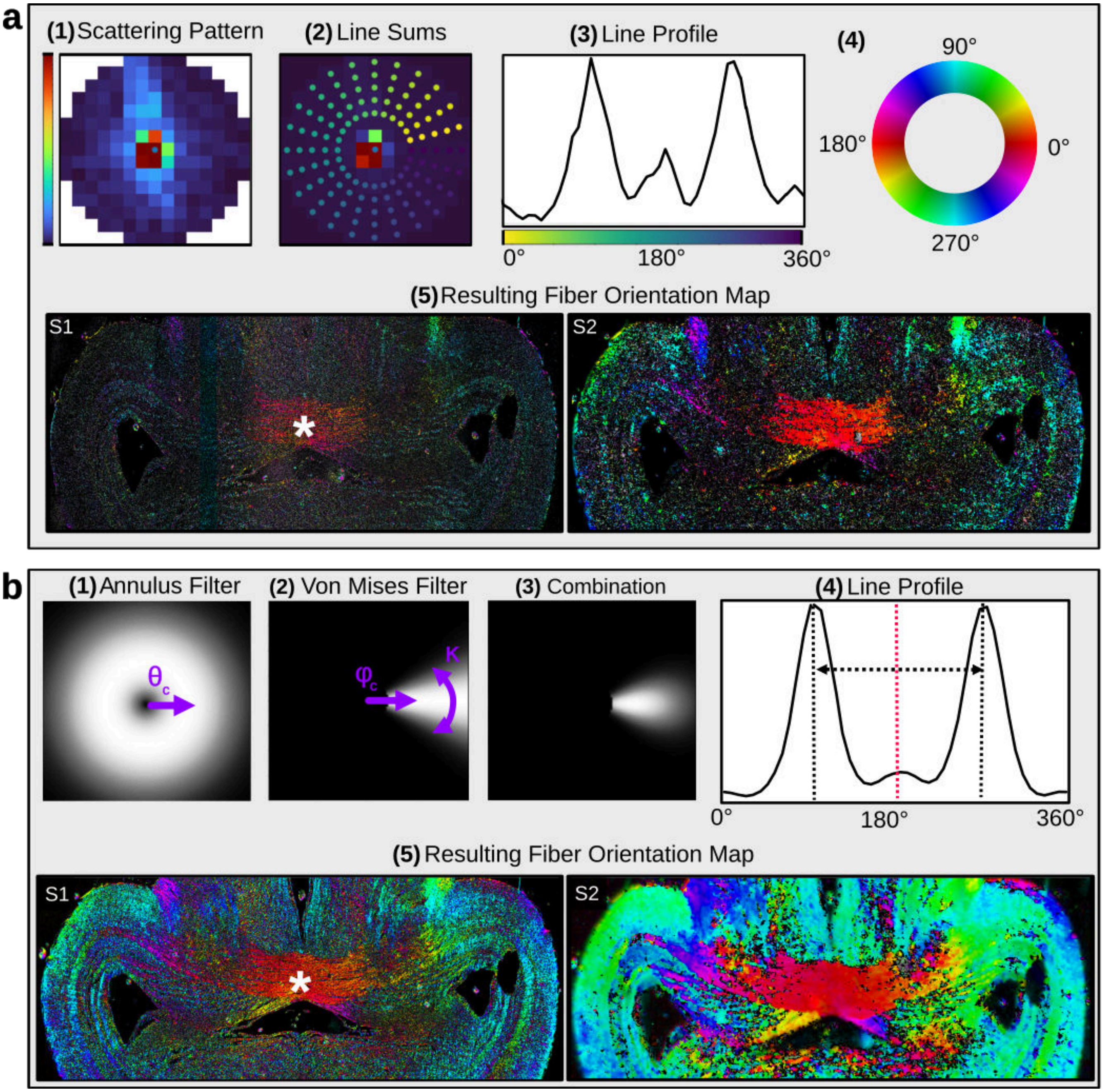

Supplementary Figure 4: Computation of ComSLI fiber orientation maps without filtering (a) and with filtering (b) of scattering patterns. a) Without filtering, each scattering pattern (1) is sampled from an inner to an outer circle along a line in 15° azimuthal steps as shown in (2), and for each azimuthal angle the sum of intensities is plotted (3). The fiber orientation is then computed from the mid-position of a peak pair in the line profile and represented by a color according to the color wheel (4). This procedure is performed for each pixel, resulting in a fiber orientation map (5), which is shown here exemplary for the mid region of the frog brain section for setup S1 and S2. b) With filtering, an annulus filter (1) and von Mises filter (2) are combined (3) and applied to the scattering pattern for the different azimuthal angles, resulting in a smoothed line profile (4). In this profile, it is much easier to reliably determine the position of prominent peaks (black dashed lines) and their mid-position (red dashed line) corresponding to the fiber orientation in the pixel, here a horizontal orientation encoded in red in the fiber orientation map (5), marked by the asterisk.

bioRxiv preprint doi: https://doi.org/10.1101/2024.11.28.625428; this version posted April 9, 2025. The copyright holder for this preprint (which was not certified by peer review) is the author/funder, who has granted bioRxiv a license to display the preprint in perpetuity. It is made available under aCC-BY 4.0 International license.

Supplementary Table 1: Histogram metrics of main Figure 2 (in degrees), showing the difference between ComSLI fiber orientations obtained from setup S1 and setup S2: mean, mode, full width at half maximum (FWHM), and root mean square error (RMSE).

	Mean	Mode	FWHM	RMSE
Mouse knee (muscle)	1.75	1.08	18.36	24.95
Mouse knee (tendon)	2.17	1.80	17.64	24.09
Mouse knee (cartilage)	3.52	-6.48	70.92	33.95
Mouse knee (bone)	-0.52	22.32	94.32	40.64

Supplementary Table 2: Histogram metrics of main Figure 5 (in degrees), showing the difference between ComSLI and STA fiber orientations for setup S1 and S2: mean, mode, full width at half maximum (FWHM), and root mean square error (RMSE).

	Mean	Mode	FWHM	RMSE
Frog brain (S1)	0.28	-2.88	79.56	40.48
Frog brain (S2)	5.90	-2.16	60.84	25.80
Mouse knee (S1)	-6.21	-8.64	24.48	36.38
Mouse knee (S2)	0.99	-3.24	30.96	32.43
Vervet brain (S2)	-5.82	-6.12	53.64	27.11
